# A Pilot Cross-Sectional Study to Investigate the Biomarker Potential of Phosphorylated Neurofilament-H and Immune Mediators of Disability in Patients With 5 Year Relapsing-Remitting Multiple Sclerosis

**DOI:** 10.3389/fneur.2019.01046

**Published:** 2019-10-09

**Authors:** María Inés Herrera, Rodolfo Alberto Kölliker-Frers, Matilde Otero-Losada, Santiago Perez Lloret, Macarena Filippo, Julia Tau, Francisco Capani, Andrés M. Villa

**Affiliations:** ^1^Instituto de Investigaciones Cardiológicas, National Research Council, Universidad de Buenos Aires (ININCA, UBA-CONICET), Buenos Aires, Argentina; ^2^Centro de Investigaciones en Psicología y Psicopedagogía, Pontificia Universidad Católica Argentina (CIPP, UCA), Buenos Aires, Argentina; ^3^Facultad de Farmacia y Bioquímica, Universidad Maimónides, Buenos Aires, Argentina; ^4^Laboratorio de Investigación Ocular, Departamento de Patología, Facultad de Medicina, Universidad de Buenos Aires, Buenos Aires, Argentina; ^5^Departamento de Biología, Universidad John F. Kennedy, Buenos Aires, Argentina; ^6^Facultad de Psicología, Pontificia Universidad Católica Argentina UCA, Buenos Aires, Argentina; ^7^Instituto de Ciencias Biomédicas, Facultad de Ciencias de la Salud, Universidad Autónoma de Chile, Santiago, Chile; ^8^Seccion de Neuroinmunología Clínica, Servicio de Neurología, Hospital José María Ramos íMejía, Buenos Aires, Argentina

**Keywords:** relapsing-remitting multiple sclerosis, biomarker potential, neurofilament heavy chain phosphoform, cytokines, disability progression, expanded disability status scale

## Abstract

**Objective:** To test the feasibility of conducting a full-scale project evaluating the potential value of the phosphorylated neurofilament H (pNF-H) and several cytokines as disability markers in relapsing-remitting multiple sclerosis (RRMS).

**Methods:** Twenty-four patients with 5-year RRMS evolution and eleven healthy control subjects entered the study. None of the participants had an inflammatory systemic or metabolic disease. Disability progression was evaluated using the Expanded Disability Status Scale. Serum level of pNF-H, the anti-inflammatory cytokine transforming growth factor-β 1 (TGF-β1), and the pro-inflammatory cytokines tumor necrosis factor-α (TNF-α), interleukin-1β (IL-1β), interleukin-6 (IL-6), interleukin-8 (IL-8), interleukin-17A (IL-17A), monocyte chemotactic protein-1 (MCP-1), and soluble intercellular cell-adhesion molecule 1 (sICAM-1) were quantified using enzyme-linked immunosorbent assay (ELISA).

**Results:** The patients had higher serum level of TGF-β1, IL-6, sICAM-1, and pNF-H. Based on these findings, a sample of at least 49 controls and 89 recent-onset RRMS patients is required to find an at least 1-point between-group difference in pNF-H with a power of 80% and an α error = 0.05. The progression of the disease was correlated with the level of pNF-H (Spearman rho = 0.624, *p* = 0.006), but not with the cytokines'.

**Conclusions:** The serum level of pNF-H, EDSS score-correlated, might stand for a potential biomarker of disability in RRMS reflecting progressive axonal damage and cumulative neurological deterioration. The novelty of these results warrants conducting a larger confirmatory trial.

## Introduction

Multiple sclerosis is the most prevalent chronic autoimmune disease of the central nervous system with the first clinical symptoms typically appearing in early adulthood, and affecting over 2 million people worldwide (1). Relapsing-remitting multiple sclerosis (RRMS) is the current criteria-based most prevalent form of the three clinical presentations of multiple sclerosis (MS), relapsing-remitting, converted to secondary progressive, and primary progressive. It is characterized by neurologic worsening attacks or relapses followed by apparent recovery periods of masked progression of the disease (2).

The identification of a reliable biomarker of the progressive axonal degeneration is critical to prevent long-term disability in RRMS. Biomarker dynamics in blood and CSF are poorly characterized in RRMS (3), and serum levels of the neurofilament heavy chain phosphoform (pNF-H) might provide surrogate information about ongoing neurodegeneration in RRMS (4). Likewise, the differential expression of cytokines directly involved in RRMS pathogenesis might also indicate disease status. However, their biomarker potential (5) has been validated for application in clinical practice only in a few cases. We conducted a pilot cross-sectional study to investigate the biomarker potential of pNF-H and several cytokines for disability in patients with 5-year RRMS evolution. The control group included age-matched healthy subjects. Provided the purported biomarkers reflected the patients' disability degree, their detection in blood might promise useful for early diagnosis, and monitoring of the therapeutic response.

Being RRMS an orphan disease makes recruiting large patient samples difficult, time-consuming, and expensive. Therefore, given the clinical relevance of this topic, we resorted to carrying out the present pilot, preliminary small-case study, to explore the feasibility of conducting a large-scale trial to compare the levels of the pNF-H and cytokines in patients with RRMS and healthy controls using the present protocol, based on the coming out information (6–8).

## Methods

### Study Design

An observational cross-sectional, transverse analysis was conducted (2009–2012). The ethics committees of the Faculty of Medicine, University of Buenos Aires (UBA) and the José María Ramos Mejía Hospital (JMRMH) approved the protocol. All participants were informed and gave written consent.

### Subjects

Twenty-four RRMS drug-naive patients with a 5-year disease evolution and 11, age-matched healthy controls (age range 30–60 y. o.) completed a validated questionnaire on personal characteristics, medical history, smoking, diet, alcohol consumption, and current medication. Blood samples were obtained during the 3-month relapse period when the pNF-H concentration is expected to increase. The control group underwent neurological and laboratory examination at the Neurology Service of the JMRMH for inclusion purposes only.

### Inclusion Criteria for Patients

- Patients over 18 years old with a 5-year RRMS evolution and early diagnosis of the disease according to the McDonald criteria.
- EDSS value <5.5.

### Inclusion Criteria for Controls

- Healthy volunteers over 18 years old.

### Exclusion Criteria for Patients and Controls

- History of major psychiatric disorders.
- Pregnancy or lactation.
- Any systemic disease: current infection or chronic disease, autoimmune diseases except for RRMS in the patients, atherosclerosis, diabetes, metabolic syndrome, etc.

### Sampling

Blood samples were collected from patients (during remissions) and controls after 12 h overnight fasting. Blood samples were left at room temperature for 30 min until clot formation, and sera were separated and stored at −20°C until determinations. All participants underwent magnetic resonance imaging (MRI) recording.

### Neurofilament Heavy Chain Phosphoform (pNF-H) Determination

A specific sandwich ELISA kit (NS170, Chemicon International, USA-CANADA) was used to quantify pNF-H. A special ELISA 96-well chicken anti- pNF-H polyclonal antibody pre-coated immunoplate was used to capture pNF-H which was detected using rabbit anti- pNF-H polyclonal antibody (1:100), followed by an alkaline phosphatase-conjugated (1:2000) goat anti-rabbit polyclonal antibody. After pNPP (p-nitrophenyl phosphatase) alkaline phosphatase substrate addition, pNF-H was quantified by absorbance at 405 nm. Limit of detection (LOD) 0.0585 ng/mL, range of quantitation (ROQ): 0.0293 ng/mL-15 ng/mL.

### Pro-inflammatory and Anti-inflammatory Molecules Determination

To rule out traditional cardiovascular risk markers (CVRM), ELISA kits allowed quantifying the cytokines TNF-α, IL-1β, IL-6, IL-8, IL-17A, and TGF-β1 (respectively 555212, 557953, 555220, 555244, Platinum, and 559119 BD Biosciences, New Jersey, USA), and sICAM-1 and MCP-1 (respectively Human Quantikine sICAM-1/CD54 and Human Quantikine MCP-1 R&D Systems, Minneapolis, USA). LOD: 7.8 pg/mL for TNF-α, 3.9 pg/mL for IL-1β, 4.7 pg/mL for IL-6, 3.1 pg/mL for IL-8, 1.6 pg/mL for IL-17A, 4.7 pg/mL for TGF-β1, 1.56 ng/mL for sICAM-1, and 2.31 pg/mL for MCP-1.

In all cases, ELISA kits were used according to technical instructions and following technical specifications.

#### TNF-α Assay Kit

The Human TNF-α (555212), ELISA kit (BD Biosciences, New Jersey, USA) is an *in-vitro* ELISA for the quantitative measurement of human TNF-α in sera. The lower limit of detection is 7.8 pg/mL (the lowest positive standard value).

#### IL-1β Assay Kit

The Human IL-1β (557953), ELISA kit (BD Biosciences, New Jersey, USA) is an *in vitro* ELISA for the quantitative measurement of human IL-1β in sera. The lower limit of detection is 3.9 pg/mL (the lowest positive standard value).

#### IL-6 Assay Kit

The Human IL-6 (555220), ELISA kit (BD Biosciences, New Jersey, USA) is an *in-vitro* ELISA for the quantitative measurement of human IL-6 in sera. The lower limit of detection is 4.7 pg/mL (the lowest positive standard value).

#### IL-8 Assay Kit

The Human IL8 (555244) ELISA kit (BD Biosciences, New Jersey, USA) is an *in-vitro* ELISA for the quantitative measurement of human IL-8 in sera. The lower limit of detection is 3.1 pg/mL (the lowest positive standard value).

#### IL-17A6 Assay Kit

The Human IL-17A Platinum ELISA kit (BD Biosciences, New Jersey, USA) is an *in vitro* ELISA for the quantitative measurement of human IL-17A. The lower limit of detection is 1.6 pg/mL (the lowest positive standard value).

#### TGF-β1 Assay Kit

The Human TGF-β1 (559119), ELISA kit (BD Biosciences, New Jersey, USA) is an *in vitro* ELISA for the quantitative measurement of human TGF-β1 in sera. The lower limit of detection is 4.7 pg/mL (the lowest positive standard value).

#### sICAM-1 Assay Kit

The Human Quantikine sICAM-1/CD54 ELISA kit (R&D Systems, Minneapolis, United States of America) is an *in-vitro* ELISA for the quantitative measurement of human sICAM-1 in sera, plasma and cell culture supernatants. Sera were used to determine sICAM-1 levels according to technical specifications. The lower limit of detection is 1.56 ng/mL (the lowest positive standard value).

#### MCP-1 Assay Kit

The Human Quantikine MCP-1 ELISA kit (R&D Systems, Minneapolis, United States of America) is an *in-vitro* ELISA for the quantitative measurement of human MCP-1 in sera, plasma and cell culture supernatants. Sera were used to determine MCP-1 levels according to technical specifications. The lower limit of detection is 2.31 pg/mL (the lowest positive standard value).

### Disability Assessment Using the Expanded Disability Status Scale (EDSS)

The worldwide used Expanded Disability Status Scale (EDSS) (9) for assessing neurological impairment and disability progression evaluates:

- Pyramidal weakness or difficulty in moving limbs;
- Cerebellar ataxia;
- Loss of coordination or tremor;
- Brainstem problems with speech, swallowing, and nystagmus;
- Sensory numbness or loss of sensations;
- Bowel and bladder dysfunction;
- Visual dysfunction;
- Mental dysfunction.

Scale grading: 0 = normal neurological exam, 1.0–4.5 = ambulatory patients, 5.0–9.5 = impaired ambulation, and 10 = death by RRMS (8).

### Statistical Analysis

Non-parametric tests were used due to the non-Gaussian data distribution and the small sample size. The Mann-Whitney *U*-test yielded between-groups comparison, and the Spearman's Rho method evaluated correlations. Assessment of possible differences between patients native or referred to our tertiary center was performed by logistic regression. The alpha error was set at 0.05 (SPSS 23.0, NY, USA). The reduced number of cases studied precluded us from conducting a multivariate analysis to discard potential confounding factors. All the same, controls and patients were fairly indistinguishable as for gender and age.

## Results

Eleven healthy controls and 24 RRMS patients were recruited (43 and 39% of males respectively, *p* = 0.645). Median (25th; 75th percentiles) age was 38 years old (33; 53) for controls and 39 years old (31; 43) for patients (*p* = 0.699). The patients' group had a median EDSS score of 2.3 (2.0; 3.5), minimum and maximum scores were 1 and 6. Five patients were referred from other centers.

**Figure 1:**
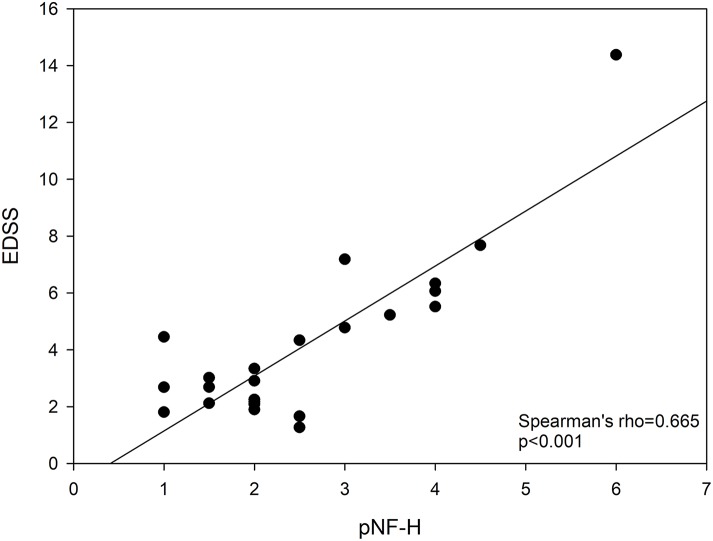
Relationship between EDSS and pNF-H. EDSS, Expanded Disability Status Scale; pNF-H, phosphorylated neurofilament H.

The RRMS patients had higher plasmatic levels of pNF-H, TGF-β1, IL-6, and sICAM-1 (Table 1). In RRMS patients, EDSS score was not related with IL-17A (rho = 0.094, *p* = 0.760, *n* = 13), TGF-β1 (rho = 0.052, *p* = 0.880, *n* = 11), IL-1β (rho = −0.114, *p* = 0.711, *n* = 13), TNF-α (rho = −0.168, *p* = 0.583, *n* = 13), IL-6 (rho = 0.125, *p* = 0.683, *n* = 13), IL-8 (rho = −0.316, *p* = 0.293, *n* = 13), MCP-1 (rho = 0.274, *p* = 0.476, *n* = 9), and sICAM-1 (rho = 0.170, *p* = 0.688, *n* = 8). Conversely, pNF-H correlated with EDSS score (rho = 0.624, *p* = 0.006, *n* = 18, Figure 1). A logistic regression analysis ruled out any confounding effect due to the patients' referring center (*p* = 0.705).

**Table 1:** Neuroimmune markers in RRMS patients and healthy controls.

	**Controls (*n* = 11)**	**RRMS patients (*n* = 24)**	***p*-value**
pNF-H	0.16 (0.15; 0.18)	3.17 (2.13; 5.37)	**<0.001**
IL-17A	5.44 (4.22; 8.20)	8.28 (6.77; 9.72)	0.055
TGF-β1	80.00 (39.00; 150.00)	1087.00 (557.00; 2,417.00)	**<0.001**
IL-1β	4.30 (2.70; 10.53)	7.38 (3.64; 15.77)	0.296
TNF-α	3.00 (1.56; 6.00)	4.33 (1.67; 8.78)	0.497
IL-6	7.47 (3.87; 9.00)	21.00 (14.93; 35.46)	**<0.001**
IL-8	8.89 (4.12; 13.70)	18.63 (11.70; 41.00)	**<0.019**
MCP-1	131.00 (108.00; 157.63)	162.00 (112.63; 211.00)	0.207
sICAM-1	222.04 (212.78; 252.90)	288.70 (246.11; 444.00)	**0.015**

*Values are shown as median (25th; 75th percentiles)*.

## Discussion

We have observed a median 3-point difference in pNF-H value between RRMS patients and healthy controls. Heterogeneity regarding disease severity due to the contribution of mild to intermediate RRMS patients in this study might account for data dispersion and contribute to concealing between-group differences. To better understand the value of determining serum pNF-H level, a clinical study should include at least 49 controls and 89 recent-onset RRMS patients to find an at least 1-point between-group difference in pNF with a power of 80% and an α error = 0.05, based on the variability parameters calculated in this study. Our study showed a correlation between the pNF-H level and disease progression, but multivariate analyses to identify possible confounding factors were not possible due to the reduced number of cases. Typically, these studies require 200–300 patients with varying disease evolution.

Unlike inflammatory cytokines, the serum level of pNF-H (axonal damage) correlated with EDSS score values, suggesting its likely reliability in monitoring the clinical condition in RRMS patients with 0–5.5 EDSS score values.

A study by Trapp and colleagues showed that axonal pNF-H staining was more frequent in MS patients than in healthy controls (10). Thereafter, pNF-H has been proposed as a surrogate marker of axonal injury in many neurodegenerative diseases (11). In MS, while cerebrospinal fluid or blood phosphorylated light chain neurofilament level has been proposed as a useful clinical biomarker (12, 13), results on pNF-H are scarce. Patients with a high pNF-H level are exposed to an evolutionary disability risk higher than those with high TGF-β1, IL-6, and sICAM-1, which showed a poorer relationship with disability.

Cytokines reflect an inflammatory state, which makes up part of a complex process of neuronal death (14). The lack of correlation between cytokine levels and EDSS-score reinforces the idea that no single cytokine has so far emerged as an undisputed biomarker candidate of disability state though they should be confirmed in a larger number of patients. Regardless of the unquestionable immunopathogenic role of cytokines in RRMS, their fluctuations add complexity to the inherent uncertainty derived from inflammatory outbreaks and remissions over time. Idiosyncrasy concerning inflammation characteristics and the individual response to treatment precludes cytokines from embodying a good marker of evolutionary risk. The increase in TGFβ-1 level in RRMS patients agrees with the clinical remission phase they were at the time of blood sampling.

Petzold (15) reported an increase in pNF-H level in patients with progressive disease. The positive correlation found between serum pNF-H and the EDSS score in patients with a 5-year RRMS evolution in this study may well serve as the cornerstone on the way to validation.

The outcome of this pilot study provides empirical evidence to put forward pNF-H and certain cytokines as likely RRMS biomarkers. Keeping in mind Carl Sagan' statement on absence of evidence is not evidence of absence, noteworthy the serum level of pNF-H (axonal damage) unlike serum cytokines (inflammation) correlated with EDSS score. Our data show that determination of pNF-H and cytokines complement each other in RRMS. Currently, we pursue developing a neuroinflammation score increasing their conjoint predictive power.

## Conclusion

A large clinical study to better understand the value of the pNF as a prognostic, early marker of MS disability progression is feasible using the currently used protocol. Likewise, it may aid the neurologist in customizing the treatment. Based on the variability parameters observed in this study, it should include at least 49 controls and 89 recent-onset RRMS patients to find an at least 1-point between-group difference in pNF with a power of 80% and an α error = 0.05. A suitable full-scale multicenter study should conveniently evaluate both early diagnosed and advanced-stage RRMS patients, with a thorough evaluation of the disease, and follow-up if possible.

## Limitations of This Study

This pilot study evaluated a small sample of patients. This resulted in a lack of statistical power, inability to conduct multivariate analyses, and poor generalizability beyond the reduced number of cases studied.

## Data Availability Statement

The datasets generated for this study are available on request to the corresponding author.

## Ethics Statement

The ethics committees of the Faculty of Medicine, University of Buenos Aires and the José María Ramos Mejía Hospital approved the protocol. All participants were informed and gave written consent.

## Author Contributions

MH: writing. RK-F: original idea and investigation. MO-L: review and editing—grammar, style, and language. SP: statistical analysis, table, and figures. MF and JT: investigation. FC: funding acquisition and supervision. AV: investigation and supervision.

### Conflict of Interest

The authors declare that the research was conducted in the absence of any commercial or financial relationships that could be construed as a potential conflict of interest.
